# Correction to: Sunitinib-suppressed miR-452-5p facilitates renal cancer cell invasion and metastasis through modulating SMAD4/SMAD7 signals

**DOI:** 10.1186/s12943-022-01568-y

**Published:** 2022-04-06

**Authors:** Wei Zhai, Saiyang Li, Jin Zhang, Yonghui Chen, Junjie Ma, Wen Kong, Dongkui Gong, Junhua Zheng, Wei Xue, Yunfei Xu

**Affiliations:** 1 Department of Urology, Renji Hospital, School of Medicine in Shanghai Jiao Tong University, 160 Pujian Road, Pudong District, Shanghai, 200127 China; 2 Department of Urology, Shanghai Tenth People’s Hospital, Nanjing Medical University, Nanjing, 211166 China; 3 Department of Urology, Shanghai Tenth People’s Hospital, School of Medicine in Tongji University, Shanghai, 200072 China; 4 Department of Urology, Shanghai First People’s Hospital, School of Medicine in Shanghai Jiao Tong University, Shanghai, 200080 China

**Correction to: Mol Cancer 17, 157 (2018)**

**https://doi.org/10.1186/s12943-018-0906-x**

Following publication of the original article [1], a minor error was identified in the images presented in Fig. 1; specifically:

Fig. 1F: incorrect image used for representative image of invasive cells induced by transfection of miR-452-5p in OSRC-2 with treatment of 10 μM Sunitinib (top left image); the image has been corrected.

The corrected figure is given here. The correction does not have any effect on the results or conclusions of the paper.

**Fig. 1:**
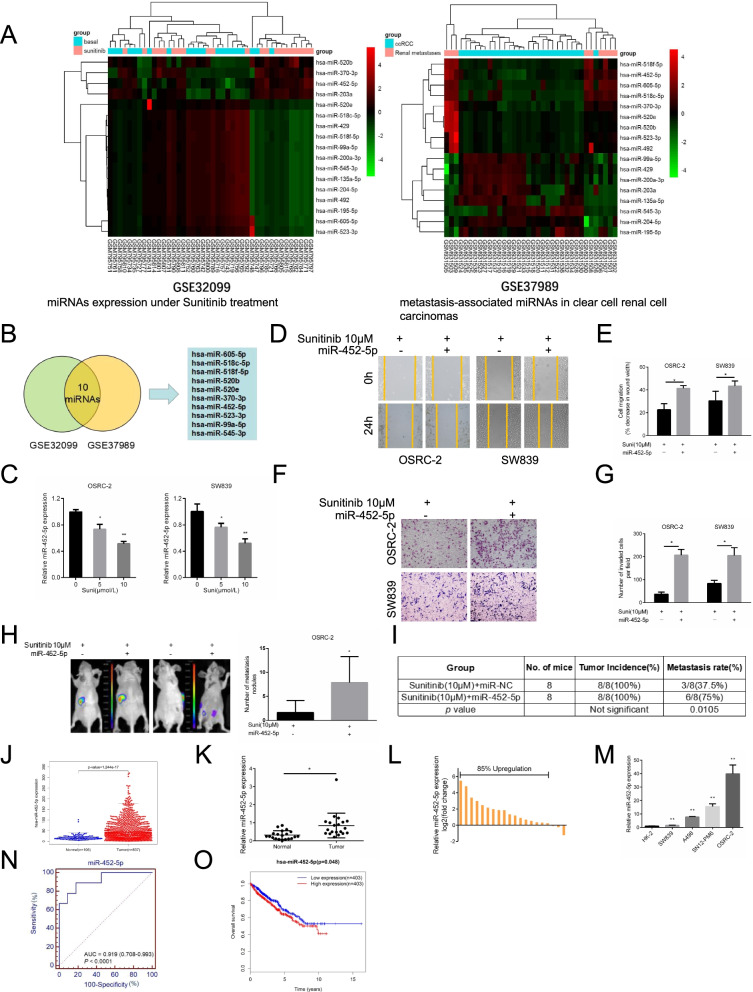
Sunitinib abrogates RCC cell invasion and metastasis via depressing miR-452-5p. **a** Presented are heatmap of the most differentially expressed miRNAs in peripheral blood under Sunitinib treatment (GSE32099) and between tumor tissue and pair-matched normal tissues(GSE37989). **b** The above analysis showed 10 miRNAs were significantly differentially expressed both in GSE32099 and GSE37989. **c** qRT-PCR assays for miR-452-5p expression with 0, 5 and 10 μM Sunitinib treatment in OSRC-2 and SW839 cells. **d**-**e** Representative micrographs of wound-healing assay and decrease in wound width was induced by transfection of miR-452-5p in OSRC-2 and SW839 cells versus miR-NC cells with treatment of 10 μM Sunitinib. **f**-**g** Representative images and number of invasive cells per high-power field was induced by transfection of miR-452-5p in OSRC-2 and SW839 cells versus miR-NC cells with treatment of 10 μM Sunitinib. **h** Orthotopic xenograft animal models were generated using miR-452-5p or miR-NC in OSRC-2 cells and mice treated with 10 μM Sunitinib. Presented are representative images (left) of abdominal metastasis viewed by IVIS system in each group 4 weeks after the orthotopic xenograft transplantation (*n*=8) and Quantitation of metastasis nodules shown at right. **i** Incidence of metastasis in orthotopic xenografts after 4 weeks. **j** TCGA cohort analysis of the differentially expressed levels of miR-452-5p in RCC tumor samples and pair-matched normal tissues. Each dot represents one sample. **k** Comparison of miR-452-5p expression in 20 paired RCC tumor tissues and adjacent normal tissues via qRT-PCR. **l** Relative miR-452-5p expression levels in RCC samples are presented as fold change=2(∆Ct normal-∆Ct tumor) of tumor versus matched normal tissues, 85% of which was upregulated in tumor tissues than adjacent normal tissues. **m** miR-452-5p expression in a series of RCC cell lines (SW839, A498, SN12-PM6 and OSRC-2) and human normal renal tubular epithelial cell line HK-2. **n** ROC analysis to assess the specificity and sensitivity of miR-452-5p to differentiate between RCC and normal tissues. **o** Kaplan–Meier analyses of the correlations between miR-452-5p expression level and overall survival of 806 patients with RCC through TCGA analysis. The median expression level was used as the cutoff. Data shown are mean±S.D. *p<0.05, **p<0.01

## References

[1] Zhai W, Li S, Zhang J (2018). Sunitinib-suppressed miR-452-5p facilitates renal cancer cell invasion and metastasis through modulating SMAD4/SMAD7 signals. Mol Cancer.

